# Vaccinium and Variolinum

**Published:** 1893-10

**Authors:** John Hall

**Affiliations:** Victoria, B. C.


### VACCINIUM AND VARIOLINUM.

VICTORIA, B. C., August 12th, 1893.

EDITOR OF THE HOMŒOPATHIC PHYSICIAN :—In my article on vaccination, which I had hoped would be printed before the last meeting of our men, there is a very important misprint in July number, on page 393, line fourteen from the bottom, where the word fully is called "fondly," an error which alters the whole tenor. Will you please call attention to this.

Sincerely,

JOHN HALL.

